# Giving an account of patients' experience: A qualitative study on the care process of hip and knee osteoarthritis

**DOI:** 10.1111/hex.13468

**Published:** 2022-03-09

**Authors:** Simone Battista, Mattia Manoni, Andrea Dell'Isola, Martin Englund, Alvisa Palese, Marco Testa

**Affiliations:** ^1^ Department of Neurosciences, Rehabilitation, Ophthalmology, Genetics, Maternal and Child Health University of Genova Savona Italy; ^2^ Department of Clinical Sciences, Clinical Epidemiology Unit, Orthopedics Lund University Lund Sweden; ^3^ Department of Medical Sciences, School of Nursing University of Udine Udine Italy

**Keywords:** clinical governance, osteoarthritis, phenomenology, physical therapists, physical therapy specialty, policy making, practice guidelines as topic

## Abstract

**Introduction:**

Despite the publication of clinical practice guidelines, the quality of the care process as experienced by patients with osteoarthritis (OA) appears suboptimal. Hence, this study investigates how patients with OA experience their disease and care process, highlighting potential elements that can enhance or spoil it, to optimise their quality of care.

**Methods:**

A qualitative study based on semi‐structured interviews. Patients with hip and knee OA in Italy were interviewed. The interview guide was created by a pool of health professionals and patients. The interviews were analysed through a theme‐based analysis following a philosophy of descriptive phenomenological research.

**Results:**

Our analysis revealed seven main themes: (1) *Experiencing a sense of uncertainty*, as interviewees perceived treatment choices not to be based on medical evidence; (2) *Establishing challenging relationships with the self and the other*, as they did not feel understood and felt ashamed or hopeless about their condition; (3) *Being stuck in one's own or the health professionals' beliefs about the disease management*, as a common thought was the perception of movement as something dangerous together with a frequent prescription of passive therapies; (4) *Dealing with one's own attitudes towards the disease; Understanding* (5) *the barriers to and* (6) *the facilitators of the adherence to therapeutic exercise*, which revolve around the therapy cost, the time needed and the patients' willingness to change their life habits and (7) *Developing an uneasy relationship with food* since the diet was considered as something that “you force yourself to follow” and overeating as a way “to eat your feelings”.

**Conclusion:**

The lack of clear explanations and a negative attitude towards first‐line nonsurgical treatments (mainly physical exercise), which are considered as a way to fill the time while waiting for surgery, underlines the importance of providing patients with adequate information about OA treatments and to better explain the role of first‐line intervention in the care of OA. This will enhance patient‐centred and shared decision‐making treatments.

**Patient Contribution:**

Patients with hip and knee OA participated in creating the interview and contributed with their experience of their care process.

## 1 INTRODUCTION

The care process is often a complex and intimate experience as lived by patients. Caring is one of the expressions of the ontology of human beings since people are dependent on one another.^1^, ^2^ Due to its grounded origin in anthropology, the care process can be considered as a ‘ritual’ performed around the patient.^3^ It is a complex psychosocial context with a nonspecific effect on patients' brain that can amplify or reduce the specific effect of the treatment, tapping into people's beliefs, expectations and feelings.^3^, ^4^, ^5^ An example of this complexity can be found in the care of individuals with hip and knee osteoarthritis (OA).

OA is the most common form of arthritis and one of the foremost causes of compromised health‐related quality of life worldwide.^6^, ^7^ Its first‐line intervention includes therapeutic exercises and education programmes, often delivered by physiotherapists, and diet when needed.^8^, ^9^, ^10^, ^11^ Therefore, this intervention is grounded on highly demanding nonsurgical treatments in terms of patients' compliance and on a cultural change at the individual and societal levels that need to accept and foster the importance of these interventions.^12^, ^13^

To answer this need, several national and international clinical practice guidelines (CPGs) were published all over the world.^8^, ^9^, ^10^, ^11^ However, different international studies brought to the forefront that only one‐third of patients are receiving the recommended first‐line interventions.^14^, ^15^, ^16^ Basedow and Esterman^14^ analysed the appropriateness of OA care through the synthesis of quality indicators retrieved from global medical records, administrative databases and patient questionnaires and interviews. These indicators are generally expressed as pass‐rates, and they concluded that the quality of OA care was suboptimal for all treatment domains, with first‐line interventions reaching the lowest pass‐rate.^14^

The reasons behind the failure of the implementation of first‐line interventions are several. They include, among others, patients' preferences, beliefs and experiences about their disease and the care process behind it.^4^ In the healthcare setting of musculoskeletal conditions, such as OA, efforts on assessing and understanding patients' experience could help health policy‐makers foster improvements in healthcare providers' interpersonal aspects and patients' expectations on how healthcare should be delivered.^17^

Therefore, it is essential to investigate the experience of people with OA about their disease, focussing on the care process they received, exploring their experiences, preferences and beliefs, so as to highlight potential elements that affect it, both positively or negatively. For what concerns the Italian healthcare system, it provides its citizens with universal coverage essentially free of charge.^18^ However, Italy lacks national CPGs for OA management that are officially recognised by the Italian Higher Institute of Health. This results in the absence of a standardised care process for this disease. Therefore, health professionals can only rely on international CPGs, which we showed elsewhere to be scarcely followed by Italian physiotherapists.^19^ Hence, this qualitative study explored the experience of people with OA about the care process they received in Italy.

## 2 METHODS

### 2.1 Study design

A descriptive phenomenological study was performed from October 2020 to March 2021. The descriptive phenomenological inquiry aims at ensuring “direct explorations, analysis and descriptions of particular phenomena (as in this case—the care process), as free as possible from unexamined presuppositions, maximising intuitive presentation”.^20^, ^21^, ^22^, ^23^, ^24^ The underpinning intent of the phenomenological researcher is to give voice and power to individuals who experienced, or are experiencing, at first‐hand the phenomenon of interest as, in this instance, people with OA.^22^

The research was conducted in respect of the Declaration of Helsinki and reported following the Consolidated Criteria for Reporting Qualitative Research (COREQ; Table S1).^25^ Ethical approval was obtained from the Ethics Committee for University Research (CERA: Comitato Etico per la Ricerca di Ateneo), University of Genova (Approval date: 15 June 2020; CERA2020.07).

### 2.2 Participants

A purposeful sampling method was adopted to ensure the maximum variations of the experiences.^26^ Participants living in different geographic locations, both urban (core areas of cities) and suburban (residential areas that surround main cities), with hip OA, knee OA or hip and knee OA, were considered eligible according to the need to reach a broad understanding of the studied phenomenon, which stems from different experiences.^26^ Specifically, individuals with physician‐diagnosed knee and hip OA, able to speak Italian and willing to participate were considered eligible to join this study. Those patients reporting joints other than hip or knee as the primary joints for OA symptoms were not considered eligible.

A network, including physicians (i.e., orthopaedics, rheumatologists and general practitioners) and other health professionals (i.e., physiotherapists and nurses), specialised in the rehabilitation of rheumatic and musculoskeletal diseases was created to help with the participants' recruitment. Health professionals were first approached individually by the research team and informed about the study aims and procedures of data collection. After obtaining their collaboration, the eligible participants were contacted by the health professionals in the network and informed on the aim of the study, the interview process (i.e., places and proposed dates) and the data confidentiality and anonymity. Eligible participants were left free to join the research and to withdraw from it at any time. Only those who expressed their interest in partaking in the study were contacted by S. B. to collect the informed consent and arrange the interview. Then, a snowball sampling was also adopted to access individuals in the network of participants with OA who had been contacted and agreed to participate first.^26^ The recruitment was concluded once the data saturation was reached, as judged by the two authors (S. B. and M. M.) who analysed interviews. An inductive thematic saturation was followed to assess the data saturation: S. B. and M. M. kept interviewing and analysing the interviews simultaneously until no new themes were found.^24^

### 2.3 Data collection method

A semi‐structured interview was designed based on the existing literature. An interview guide (Table 1) was specifically developed for this study by a pool of physiotherapists, psychologists, nurses and people with OA.^8^, ^9^, ^10^, ^11^, ^13^, ^19^, ^27^, ^28^, ^29^, ^30^ The interview guide consisted of open questions exploring different topics related to the OA care process: (a) experiential and emotional dimensions; (b) expectations and (c) beliefs. Follow‐up questions were frequently asked to investigate participants' experiences further. Examples of these questions were, ‘Can you give me an example?’ and ‘Can you explain to me what you mean with this sentence?’. At the beginning of each interview, the participants filled in the informed consent and provided their demographic (i.e., age, gender, nationality, retirement, area of living) and clinical information (i.e., height and weight to calculate body mass index, joint(s) with OA and years living with the pathology) which were registered on an electronic sheet. Only the interviewer and the interviewee were present during the interview process. No follow‐up interviews were performed.

**Table 1:** Interview guide and domains investigated

Questions	Domains
1) I would like to start this interview by asking you how you realised that you have OA?	Experiential and emotional experiences
2) How did you manage your disease?	Experiential and emotional experiences
3) What prompted you to go to a physician/health professional?	Experiential and emotional experiences
4) What did you expect from your physician/health professional the first time you saw them for OA?	Expectations
5) How were you diagnosed?	Experiential and emotional experiences
6) Would you like to tell me how you believed your disease would evolve in the future?	Beliefs
7) Did you have any family or friends that supported you during your care process?	Experiential and emotional experiences
8) Would like to tell how you felt when you received your diagnosis?	Experiential and emotional experiences
9) How would you describe the impact of OA in your life/work?	Experiential and emotional experiences
10) Would you like to tell me which treatments you expected to be suggested to manage your disease?	Expectations
11) Eventually, which treatments were in fact suggested?	Experiential and emotional experiences
12) Would you like to describe which treatments you deem useful in the management of OA?	Beliefs
12) …For example, physical activity? Manual therapy?	
13) What did you expect from the treatments that you have received so far?	Expectations
14) Would you like to tell me which roles physical activity and diet play in the management of OA?	Beliefs
15) What does a healthy diet mean for you?	Beliefs
16) Would you like to tell me the reasons why a person with OA may not be willing to change their lifestyle, integrating physical activity and a healthy diet into their daily routine?	Beliefs
17) Would you like to tell me the role of the physiotherapist in the management of OA? … And what would you expect from this professional figure?	Beliefs
18) Which attitudes did you expect from the health professionals you met during your care process towards OA? And which one(s) did they adopt?	Expectations
19) If you've ever been shown, would you like to tell me how you felt when you saw your radiographic findings?	Experiential and emotional experiences
20) In your opinion, how important were radiographic findings in your OA care process? … How important were they for the health professionals you met?	Beliefs/experiential and emotional experiences
21) Would you like to tell me how you live with OA now?	Experiential and emotional experiences
22) Is there anything else you would like to add?	Closing question

Abbreviation: OA, osteoarthritis.

The interviews were performed by S. B. and lasted approximately one hour each. S. B. is a physiotherapist and a PhD candidate trained in advanced qualitative methodologies, with proficiency in conducting qualitative studies. S. B. recognises himself as male. Participants were not aware of his professional background, and none of them had close relationships with him. The interviews were performed online, by videoconferencing, and they were conducted only with the interviewee. An audio–visual recording of each interview was produced and transcribed *verbatim* by two authors (S. B. and M. M.).

### 2.4 Data analysis

As far as the analysis was concerned, a theme‐based analysis was performed.^31^ Thematic analysis is an independent qualitative descriptive approach described as “a method for identifying, analysing and reporting patterns (themes) within data”.^32^ Since this study explored the experience of the care process, the thematic analysis was conducted within the framework of a descriptive phenomenology study, as reported and explained above.

Two authors (S. B. and M. M.) read the transcribed interviews several times to obtain a global impression of the content. S. B. and M. M. are PhD candidates (S. B. is a physiotherapist, M. M. is a psychologist), trained in qualitative methods, who both identify themselves as male. They both analysed first independently, and then jointly, the interview transcripts. Specifically, initial coding involved examining the data line by line to search for subthemes, themes, concepts and patterns. Meaning units (i.e., words, phrases and sentences that described the phenomenon of interest) were identified and framed into codes, representing significant and central aspects of the reported statements.^33^ Throughout this process, emerging codes were compared to previous codes to understand the experiences of the OA care process as lived by the participants and to generate focussed codes. Finally, the focussed codes and coding were merged and synthesised to extract final subthemes and themes. For each theme, exemplary quotes were identified and reported anonymously. The themes were derived from the data and not determined in advance.

### 2.5 Rigour and trustworthiness

To ensure the study rigour and trustworthiness, multiple strategies were promoted. Firstly, S. B. documented field notes (‘Memos’) after completing each interview to promote reflexivity.^34^ These memos were shared during research meetings for reflexive thoughts. Secondly, the research team met frequently to refine the themes and subthemes until a consensus on the final themes was achieved. Thirdly, an audit trail containing meeting notes, analysis discussions and research decisions was continuously reorganised by the two authors who analysed the interviews (S. B. and M. M.) to stress the dependability and confirmability of the study.^34^ An example of it is reported in Table 2. Lastly, a Synthesised Member Checking was exploited to improve the credibility of the analysis.^35^ At the end of each interview, participants were asked if they wanted to participate in the member checking phase. All of them agreed to partake in it. The member checking phase was yielded at the end of the interview and analysis process. The participants were provided with a one‐page summary, highlighting the main themes and subthemes identified in the study, together with a brief plain explanation of the key findings. They were then asked to read it thoroughly, feeding back the researchers with any doubts or concerns they might have had about this summary. All the participants agreed with what was retrieved, and no further modifications to the results were done.

**Table 2:** Data synthesis by extracting and abstracting findings in common themes and subthemes

Abstraction: Themes	Abstraction: Subthemes	Codes defined by researchers	Example of quotes extracted from the interviews
*Experiencing a sense of uncertainty*	*Need of a straightforward treatment*	Need of guidelines to follow	‘… Yes, I would like to have a precise guideline, also regarding nutrition… It looks as if there are some things that are left to our own intuition’. (P2, female, 68)
		Precise treatment	‘Erm. I expected them to give me clear indications on how to deal with my disease’. (P8, male, 66)
	*Doubts (treatments to follow and pathology genesis)*	Lack of understanding of the disease's mechanisms	‘They tried to explain to me how OA works somehow, but I still don't have a clear idea of how it works’. (P3, female, 73)
		Need to hear several health professionals to have a definitive answer	‘In my experience, I've had to consult two or three physicians, unless the first two agree’. (P5, female, 72)
		Need to explain the disease set‐up biomechanically	‘I thought [OA] was a consequence of bad posture, as I've been using my leg wrongly after slipping on ice once’. (P6, male, 55)
	*Different opinions heard by various health professionals*	Feeling that the physicians' decisions are not evidence‐based	‘There is an almost religious way of thinking about how to deal with the pathology. It is not an exact science; when you choose the physicians, you choose the treatment’. (P1, male, 49)
		Confusion caused by consulting different physicians	‘Maybe the fact that I did not have only one physician at the very beginning did not help me to understand how to deal with OA’. (P9, female, 73)
	*Frustration and anger*	Worrying for hearing different opinions	‘I was worried because we, as patients, hear different opinions coming from our friends and acquaintances that give us their personal point of view on how they take care of their disease’. (P9, female, 73)
		Anger/frustration for different opinions	‘It is very frustrating for a patient [not to have a precise indication] because you expect to have a disease, and a common one too, so the care process should be clear’. (P1, male, 49)
*Establishing a challenging relationship with the self and the other*	*Not being understood and the importance of empathy*	Seeing the patients as a diagnosis and not as human beings	‘There I seemed to be like… Erm… a number, a gear in the mechanism… Maybe they didn't consider me as a human being (laugh)’. (P3, female, 73)
		Lack of empathy	‘The orthopaedists did not give me much attention, and they told me that I have OA and that I have to live with it’. (P2, female, 68)
	Shame	Feeling shameful	‘I felt it [shameful] recently. I went to the beach with my granddaughter […] she wanted me to be involved in her games, and she said “Grandma come, sit down next to me”. I had to kneel down to play in the sand with her… I felt, how can I say… erm… like a piece of wood, like someone who can no longer manage their body’. (P2, female, 68)
		Limping as an unpleasant sensation	‘It is an unpleasant sensation, it feels as if you are limping. mentally though, you see? Because I do not know if it is visible or if that is only a perception [that I am limping]’. (P4, female, 47)
	Hopelessness	Hopelessness for the prognosis	‘When I received my diagnosis, they told me I had only few years left [before the surgery], and they told me “Chill and don't do anything”. I asked the second orthopaedic who visited me “If I keep on being active will I undergo surgery in 5 years instead of 10 years?” and he answered, “You are quite optimistic in both scenarios”’. (P1, male, 49)
		No possibilities to do other interventions	‘Erm. Yes [I can only do surgery]. because I dragged it on for too long and they told me that I have no other possibilities with other [nonsurgical] interventions’. (P10, male, 65)
	Use of metaphors to describe the pathology	Using a relevant metaphor/simile	‘I see it [the joint affected by OA] as a mountain which is crumbling’. (P3, female, 73)
		Associating OA to something realistic to understand it	‘My physiotherapist once told me that [OA] is like having a rusty gate, the orthopaedist decides to break it open, but if you try to grease it, it can last longer. I think that this gives you the idea’. (P1, male, 49)
*Being stuck in one's own or the health professionals' beliefs about the disease management*	Sealed faith (surgery)	Surgery as an obtrusive thought	‘It [OA surgery] is something you think about every day, something you try to resist, but that is your fate’. (P1, male, 49)
		Surgery as the final and obvious stage of OA	‘However, everyone told me: try and resist for as long as you can, but sooner or later you will have to undergo surgery, and end up under the knife, full stop’. (P10, male, 65)
	OA as a pathology of the old adults	Misperception between radiographic findings and patients' perception	‘The doctor told me: “You know that if I did not know that these x‐rays belong to you, I would think that they belong to another person who is at least 30 years older than you”… but, I guess I did not feel as bad as he was describing me’. (P11, male, 56)
		OA as an ageing process	‘They told me that I was starting to get old’. (P3, female, 73)
	Necessity of radiographic findings (diagnose/treatment)	Diagnosis only through X‐ray	‘They told me: here we have the problem, and it is evident as we can see from the x‐ray’. (P11, male, 56)
		OA as a wear and tear disease	‘Well, I had some medical check‐ups through x‐rays… and from them you could see some wear and tear joint surfaces, but they weren't uniform, nor regular, right? And so it was clear that there was something special even for a neophyte like me, right? And he [the doctor] said to me, “look, this is linked to this pathology.” And by looking at it I became aware of what my problem was’. (P6, male, 55)
	(Ab)use of passive therapies	Recommendation of ice instead of movement in young patient	‘And he [the doctor] told me that I was too young for surgery and he recommended I do this therapy, to put some ice on my joint’. (P5, female, 72)
		Recommended physical therapy for OA	‘The same rheumatologist I saw in September confirmed that the only thing I could do for my hip was Extracorporeal Shockwave Therapy’. (P4, female, 47)
	Movement as dangerous for the joint	Refrain from moving to postpone surgery	‘The doctor told me: “You have to try to postpone surgery for as long as you can. So please stop [any physical exercise]”’. (P1, male, 49)
		Avoiding exercise with a load on the joint	‘They recommended I do pilates, go swimming etc. so as to go easy on the joint, not to go to the gym, not to run etc. Basically, avoid everything that could have a violent stress on the joint at the moment’. (P4, female, 47)
*Dealing with one's own attitudes towards the disease*	Fight, resignation and acceptance	Disease as something to fight	‘From a certain perspective, I found it [OA] positive, since it is something that I have to fight against’. (P1, male, 49)
		Resignation towards getting older	‘Maybe I am accepting my becoming old, what can I say…’. (P3, female, 73)
		Acceptance of the future	‘Besides, I am also a fatalist, things happen in life and when they do, you face them’. (P4, female, 47)
	Coping strategies (primarily passive)	(Over)use of drugs	‘It's not an issue for me to take some pills not to feel any pain’. (P4, female, 47)
		Impromptu strategies to manage OA	‘I proceeded by myself with some clay, with some palliatives like unguents and things like that’. (P8, male, 66)
*Understanding the facilitators of the adherence to therapeutic exercise*	Importance of being active	Movement as an intrinsic need of the body	‘…The body has to move…’. (P3, female, 73)
		The awareness of the importance of movement	‘And, this kind of movement (walking), I realised, was good for me’. (P2, female, 68)
	Perceived exercises as concrete support to the cure	Witnessing the importance of training	‘Then the situation improved, always with training, thanks to the workouts’. (P7, female, 45)
		Difference between active and passive treatments	‘It [physical exercise] is not like taking supplements with hyaluronic acid, those (supplements) you do not see what they do’. (P1, male, 49)
	Mean to maintain functionality	The importance of movement to maintain good functionality	‘OA is difficult to cure, even impossible, it is a natural tear, only some palliatives exist. I believe that the only way, or rather, the best way is to strengthen the muscle structure so that bones and joints suffer less from the weight load on them’. (P11, male, 56)
		Movement as a way to reduce OA's impact	‘I felt well, because I kept on walking… and this allowed for reducing OA impact’. (P10, male, 65)
	Willingness to change life‐habits	The importance of determination and willpower in active care	‘…Determination and willpower [to change life‐habits]’. (P7, female, 45)
		Willpower as a compulsory step to change life habits	‘A great willpower is necessary [to change life‐habits]’. (P9, female, 73)
*Understanding the barriers to the adherence to therapeutic exercise*	Cost and lack of time	Economic consequences of the care process	‘Yes… but also from an economic point of view [it is difficult to do supervised exercises]’. (P2, female, 68)
		Being overwhelmed with life duties and forgetting about the self	‘We are trapped into a spiral in which work, we can say, takes up a lot of energy and a lot of time, and then that time is taken away from us…’. (P6, male, 55)
	Lack of clear indications	Do not know what to do with exercise	‘That is, there were some, just some things [decisions in the care process]… Erm… I don't know… they were left to our intuition, to our perception but just because you understand that by acting in a certain way, maybe you will limit its progress [of OA]…’. (P2, female, 68)
		Missing the health professionals' real intention	‘The doctor told me: “You know that if I did not know that these x‐rays belong to you, I would think that they belong to another person who is at least 30 years older than you”… but, I guess I did not feel as bad as he was describing me’. (P11, male, 65)
	Lack of willpower and fatigue in changing life habits	Losing the motivation with ageing	‘So it is that maybe when you are old, people back down, they lie on the couch,… Surely such a pain affecting someone who does not have that drive [motivation to stay fit] makes people unwilling to get up from the couch’. (P1, male, 49)
		Laziness and fatigue in changing life habits	‘I think so, for laziness. Because if you want to, you are able to find the time. So it is, therefore, laziness’. (P5, female, 72)
	Exercise perceived useful only after surgery	Exercise useful only after undergoing surgery	‘But I imagine that someone can do this… let's call it preventive activity. Activity that can help with the recovery process following the intervention’. (P6, male, 55)
		Exercise useless before surgery	‘It is useless to start doing physiotherapy/exercise if I am undertaking surgery in a month.’ (P9, female, 73)
*Developing an uneasy relationship with food*	Diet as fatigue and deprivation	Eating to eat your own feelings	‘To follow a diet is a mental fatigue […] and eating is an easy outlet to manage the stress of daily life’. (P4, female, 47)
		Diet as deprivation	‘Think about it, if someone tells you something like “From tomorrow you will eat only these things [tasteless food]”, I will only get a third of the satisfaction I normally get from eating…’. (P2, female, 68)
	Diet seen as useful only to lose weight	Diet to reduce the weight on the joints	‘Being overweight makes it worse, so obviously, the lighter I feel, the better I eat, and I also do my exercises, than [by doing so] I can see the difference’. (P7, female, 45)
		Relationship between weight and OA	‘Of course, there is a relationship [between weight and OA]. The heavier the body, the more the knee suffers, it's a matter of physics’. (P2, female, 68)

Abbreviation: OA, osteoarthritis.

### 2.6 Patient and public involvement

Two people with both knee and hip OA (one identified herself as female whilst the other one as male), representatives of two different patients associations focussed on rheumatologic and musculoskeletal diseases, were involved in the design of the study and participated in the creation of the interview guide to ensure that the questions included were relevant for the studied population.^36^ The patients' representatives did not attend the interviews and did not participate in the study.

## 3 RESULTS

Thirteen Italian people from northern Italy agreed to participate in the interviews. Two participants were not able to do the interviews because they did not understand the questions on both telephone and videoconference since they had critical auditory impairments. Therefore, eleven participants were included. Table 3 reports the sample's demographic and clinical characteristics in detail for each participant.

**Table 3:** Participants' demographic and clinical characteristics

Patient	Age	Gender	BMI	Retirement	Disease	Diagnosis
P1	49	M	26.28	No	Hip OA	X‐ray
P2	68	F	25.95	Yes	Hip and knee OA	MRI
P3	73	F	27.34	Yes	Knee OA	X‐ray
P4	47	F	28.71	No	Hip OA	MRI
P5	72	F	25.81	No	Hip OA	X‐ray and CT
P6	55	M	34.02	No	Hip and knee OA	X‐ray
P7	45	F	25.09	No	Hip OA	X‐ray and MRI
P8	66	M	28.70	Yes	Knee OA	X‐ray
P9	73	F	28.66	Yes	Knee OA	X‐ray
P10	65	M	24.69	No	Hip OA	X‐ray
P11	56	M	22.22	No	Hip and knee OA	X‐ray

Abbreviations: BMI, body mass index; CT, computed tomography; F, female; M, male; MRI, magnetic resonance image; OA, osteoarthritis; P, person.

**Figure 1:**
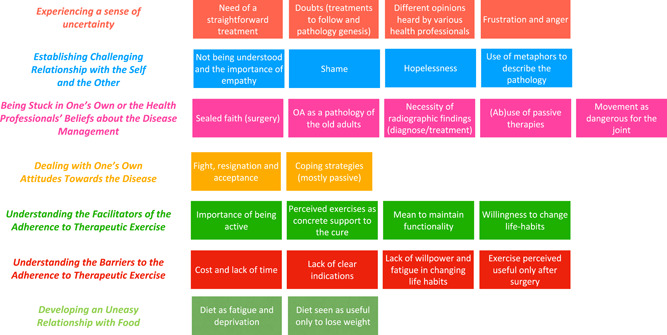
Themes and subthemes stemmed from the analysis of the interviews

Analysis of the interview data revealed seven main themes related to the OA care process (Figure 1): (1) *Experiencing a sense of uncertainty*; (2) *Establishing challenging relationships with the self and the other*; (3) *Being stuck in one's own or the health professionals' beliefs about the disease management; understanding*; (4) *Dealing with one's own attitudes towards the disease; Understanding* (5) *the facilitators of and* (6) *the barriers to the adherence to therapeutic exercise*; (7) *Developing an uneasy relationship with food*. Hereafter, the different themes that stemmed from the synthesis of their related subthemes are discussed and explored.

*Theme 1: Experiencing a Sense of Uncertainty*

A general sense of *uncertainty* regarding the care process and, precisely, how to deal with OA and which treatments should have been taken, was a transversal perception among all the interviewees. They felt that the indications they had been given were not based on evidence and that health professionals' preferences and attitudes played a crucial role in the decision‐making process of OA management.

There is an almost religious way of thinking about how to deal with the pathology. It is not an exact science; when you choose the physicians, you choose the treatment. (P1, male, age 49)

Participants were typically unaware of the causes of their disease. However, in general, they considered it to be a consequence of overuse and wrong posture, and they tried to motivate it only through a biomechanical rationale. Moreover, the explanations provided to them by the health professionals seemed fuzzy and unclear.

I thought [OA] was a consequence of bad posture, as I've been using my leg wrongly after slipping on ice once. (P6, male, 55)

They tried to explain to me how OA works somehow, but I still don't have a clear idea of how it works. (P3, female, 73)

The interviewees felt doubtful because, on the one hand, they had not received any specific indication regarding the management of OA, and, on the other, the different and not coherent opinions retrieved from the various health professionals and acquaintances increased this sense of doubtfulness and worry about their condition.

I was worried because we, as patients, hear different opinions coming from our friends and acquaintances that give us their personal point of view on how they take care of their disease. (P9, female, 73)

Besides, this sense of uncertainty led the patients to retrieve information from a plethora of different professionals, word‐of‐mouth and also from the Internet.

In my experience, I've had to consult two or three physicians, unless the first two agree. (P5, female, 72)

No, the doctors did not explain it [OA] to me. But, eventually, I looked it up on the Internet and found answers to my questions. (P8, male, 66)

As a result, they felt frustrated and angry since they wanted to receive a straightforward treatment. The lack of clear indications and the presence of the conflicting opinions heard, eventually, led them not to take care of their disease or to base their care on their intuition.

It is very frustrating for a patient [not to have a precise indication] because you expect to have a disease, and a common one too, so the care process should be clear. (P1, male, 49)

Eventually, I did not do anything anymore, just nothing. (P5, female, 72)

…Yes, I would like to have a precise guideline, also regarding nutrition… It looks as if there are some things that are left to our intuition. (P2, female, 68)

*Theme 2: Establishing Challenging Relationship with the Self and the Other*

As far the relationship with the self and the other is concerned, common feelings across the interviewees revolve around a sense of shame and hopelessness that were described with two different nuances: the former was more concerned and present in front of their beloved ones, the latter was more evoked by the lack of hope conveyed by the health professionals they consulted. Participants expressed a sense of shame caused by showing their conditions and limitations to others.

I felt it [shame] recently. I went to the beach with my granddaughter […] she wanted me to be involved in her games, and she said ‘Grandma come, sit down next to me’. I had to kneel down to play in the sand with her… I felt, how can I say… erm… like a piece of wood, like someone who can no longer manage their body. (P2, female, 68)

At the same time, they expressed a sense of hopelessness in regard to the received prognosis and the fact that physicians took for granted that this was a pathology that sees no other possibility apart from deteriorating.

Erm. Yes [I can only do surgery]. because I dragged it on for too long and they told me that I have no other possibilities with other [non‐surgical] interventions. (P10, male, 65)

Both of them were strictly related to a lack of empathy shown by both their acquaintances and health professionals. The former, since the interviewees reported that their acquaintances did not miss a chance to highlight some features related to OA (e.g., limping) that make them feel ashamed of their condition.

What annoys me the most is when people that know my condition ask me, ‘What did you do? Why are you limping?’ This makes me really upset because others see what I sometimes don't even notice. (P1, male, 49)

The latter, since the participants felt as if they were being considered as a mere number, rather than as human beings by the health professionals, they consulted, almost as if they were not worth their attention.

The orthopaedic surgeon did not give me much attention, and they told me that I have OA and that I have to live with it. (P2, female, 68)

Finally, some of the participants expressed their self‐into‐the‐pathology through the use of relevant metaphors that helped them to understand their condition better.

I see it [the joint affected by OA] as a mountain which is crumbling. (P3, female, 73)

*Theme 3: Being Stuck in One's Own or the Health Professionals' Beliefs about the Disease Management*

The participants held some core beliefs that arose after consulting with their health professionals. In particular, the most shared and common belief carried out was regarding the surgery that represented a sealed fate from which there is no escape, something that anyone with OA must eventually face.

It [OA surgery] is something you think about every day, something you try to resist, but that is your fate. (P1, male, 49)

And so when I went to see him [the physician] he said, ‘no madam, your joint is ruined… try and get on with it for as long as you can, but sooner or later you will have to do it [surgery]’. (P9, female, 73)

Another shared belief was considering OA as a disease typical of old age. Having this belief made participants feel as if they were going through an unhealthy ageing process, even if some of them felt active and alive.

The doctor told me: ‘You know that if I did not know that these x‐rays belong to you, I would think that they belong to another person who is at least 30 years older than you’… but, I guess I did not feel as bad as he was describing me. (P11, male, 56)

In fact, according to the narratives collected, health professionals have been reported to be surprised when they saw a sign of OA radiographic findings in the younger interviewees. Besides, these radiographic findings were considered necessary to diagnose OA and to decide how to plan the care process, since interviewees reported that physicians were more focussed on radiographic findings than on the symptoms they were complaining about.

They told me: here we have the problem, and it is evident as we can see from the x‐ray. (P11, male, 56)

Two other common beliefs emerged from the interviewees' encounters with their health professionals, concerned the management of OA. Firstly, the (ab)use of passive therapies that were recommended to postpone as much as possible the surgical interventions and, secondly, the low prescription of movement that was seen as dangerous as a risk factor for an anticipated surgery.

And he [the doctor] told me that I was too young for surgery, and he recommended I do this therapy, to put some ice on my joint. (P5, female, 72)

The doctor told me: ‘You have to try to postpone surgery for as long as you can. So please stop [any physical exercise]’. (P1, male, 49)

Theme 4: *Dealing with One's Own Attitudes Towards the Disease*

Interviewees showed different attitudes towards the pathology that were linked by them to their age. The older patients perceive OA as a sign of resignation towards becoming old, the younger ones as something they have to or can still fight against.

Maybe I am accepting my becoming old, what can I say… (P3, female, 73)

From a certain perspective, I took it [OA] positively since it is something I have to fight against. (P1, male, 49)

However, besides these differences, at a certain point, they all matured a sense of acceptance, as if OA is something they cannot change.

Besides, I am also a fatalist, things happen in life, and when they do, you face them. (P4, female, 47)

Both younger and older interviewees developed passive primarily coping strategies to manage their symptomatology. In particular, overuse of medications to control pain and the use of different physical therapies seemed to be the main traits of their care process, with partial room left to active therapies.

It's not an issue for me to take some pills not to feel any pain. (P4, female, 47)

I thought that by doing some thermal treatments […] mud treatments […] mesotherapy and other things… I thought that with them I would sort the disease out. (P5, female, 72)

Themes 5: *Understanding the Facilitators of the Adherence to Therapeutic Exercise*

Different facilitators of the adherence to therapeutic exercise were pointed out by the interviewees, among which the perceived benefit of doing exercises was the most shared one. In particular, being active was perceived as an intrinsic need of the body, allowing individuals to maintain a good level of functionality and as real and concrete support to the care process, something whose benefits could be seen in real‐time compared to taking medications.

…The body has to move… (P3, female, 73)

It [physical exercise] is not like taking supplements with hyaluronic acid, those (supplements) you do not see what they do. (P1, male, 49)

Moreover, all the interviewees highlighted the importance of willpower, which was seen as either a facilitator or a barrier depending on its presence or lack thereof (see next theme). All interviewees agreed that their willingness to change their life habits was the key to sticking to the exercise plan and that willpower was a compulsory step to change life habits.

…Determination and willpower [to change life‐habits]. (P7, female, 45)

Theme 6: *Understanding the Barriers to the Adherence to Therapeutic Exercise*

The barriers to the adherence to therapeutic exercise revolved around the cost of the therapy, the time needed, the lack of clear indications, the lack of willingness to change life habits and the perception of OA exercises useful only after surgery. The interviewees complained about the fact that exercise needs time, energy and money. Being absorbed in their jobs and family daily routines jeopardised their willingness to start doing physical exercises.

Yes… but also from an economic point of view [it is difficult to do supervised exercises]. (P2, female, 68)

We are trapped into a spiral in which work, we can say, takes up a lot of energy and a lot of time, and then that time is taken away from us… (P6, male, 55)

The lack of clear indications from specialists regarding the type of exercise to perform, as well as its intensity and frequency, were another barrier to starting any physical activity. The interviewees felt lost, as they perceived that they were missing precise guidelines to follow and that everything was left to their intuition.

My doctor told me to ‘go for a walk’, or maybe to ‘move’, but never specifically, something like ‘it would be better in your case to do something more targeted’. (P11, male, 56)

The interviewees highlighted that the lack of willpower and the fatigue in changing life habits were some of the main reasons behind not sticking to the exercise plan together with losing the motivation with the ageing process.

I think so, for laziness… Because if you want to, you are able to find the time. So it is, therefore, laziness. (P5, female, 72)

Finally, feeling that exercise is useful only after surgery was another barrier to sticking to the exercise plan. The interviewees perceived that it was not valuable to invest time and energy in doing exercise if surgery was their final destiny.

It is useless to start doing physiotherapy/exercise if I am undertaking surgery in a month. (P9, female, 73)

The only preventive function that exercise seemed to have was to facilitate the recovery process after the surgery. The interviewees reported little to no appreciation of exercise as a treatment in its own right.

But I imagine that someone can do this… let's call it preventive activity. Activity that can help with the recovery process following the intervention. (P6, male, 55)

Theme 7: *Developing an Uneasy Relationship with Food*

Participants had developed an unhealthy relationship with food. As a matter of fact, they perceived diet as a deprivation, a sort of sacrifice in terms of time and mental fatigue, something that they forced themselves to follow. Moreover, for some of them, overeating was being used ‘to eat your feelings’.

To follow a diet is a mental fatigue […] and eating is an easy outlet to manage the stress of daily life. (P4, female, 47)

Furthermore, when it came to the management of OA, the participants considered following a diet only as a way to reduce weight on their joints, drawing a direct relationship between weight and joint load.

Of course, there is a relationship [between weight and OA]. The heavier the body, the more the knee suffers, it's a matter of physics. (P2, female, 68)

## 4 DISCUSSIONS

The quality of the care process depends not only on the appropriateness of treatments delivered by health professionals (technical aspects) but also on relational and functional aspects.^37^ From our results, several of these aspects were hindered during the OA care process, and this could be one of the possible explanations for why patients with this disease do not reach good levels of health‐related outcomes and adherence towards first‐line interventions.

As far as the technical aspects are concerned, patients did not receive first‐line interventions. As a matter of fact, their care process was mainly based on passive therapies while waiting for surgery, with a scarce prescription of movement, seen not only as dangerous, but even as a possible risk factor for anticipated surgery, and valuable only after joint replacement. However, oral medications have been shown to reach similar effects to exercise therapy for improving functionality and pain relief in OA.^38^ Nevertheless, oral medications, and in particular nonsteroidal anti‐inflammatory drugs, have potential and well‐documented side‐effects, such as gastrointestinal toxicity, cardiovascular adverse effects and nephrotoxicity,^39^ whereas exercise has been shown to have benefits that go beyond joint health, reducing the risk of developing a wide array of comorbidities and promoting a healthy lifestyle.^40^ Moreover, evidence showed that individuals with OA are seeking non‐pharmacological and non‐surgical treatments for their conditions, and they want more information about these treatments, which highlights the likelihood that they might be open to undergoing exercise and physiotherapy interventions if educated on their benefits.^41^, ^42^ This was also retrieved in our interviews in which patients understood the importance of being active and saw exercise as a concrete support to their care, something that one can see while doing it, in contrast to medications and supplements that do not immediately show tangible effects.

According to the findings of this paper, there are several aspects that may facilitate or hinder patients' adherence towards physical exercise, and these should be taken into account by clinicians once it is prescribed. Regarding the obstacles, the lack of time and the cost seemed to be the main ones. The former was already known to be one of the more prominent causes of lack of adherence to physical exercise, in general.^43^, ^44^, ^45^ However, preliminary evidence showed that there is a gap between perceived and real lack of time^46^ and that is why it is essential to address this issue while educating the patients, to help them reach a good level of awareness of the real‐time they can devolve to their own care, and to tailor self‐management programmes according to their needs.^47^ The latter, on the other hand, has to be tackled at a higher health‐policy level. People in lower socioeconomic positions show a higher incidence of OA, more severe symptomatology and tend to experience lower benefits from OA interventions.^48^, ^49^ However, despite Italy's public healthcare system, none of the participants who were being followed by professionals within the public system reported that exercise was included in their care process. There is a need to establish an effective care pathway for people with OA, to implement evidence‐based treatments in the care routine employed in the public system, with the aim of reducing the healthcare inequalities faced by those patients who are not able to access alternative private systems.

Another barrier to the implementation of exercise into the patients' self‐management routine was their unwillingness to change their habits. This was a shared barrier with the implementation of diet strategies into their care. It is already known that individuals do not engage in health‐promoting behaviours, even though these can reduce mortality and contribute to their wellness.^50^ However, our findings show that patients were not motivated by their health professionals to follow such treatments. As reported by Hardcastle et al.,^51^ there are several strategies that professionals can implement to foster patients’ motivation, such as strategies targeting self‐efficacy, outcome expectancies, effort and value beliefs, as well as motivational interviewing techniques, and these should be used and implemented for more effective communication. Moreover, as far as the diet is concerned, it was seen by the interviewees as applicable only to reduce weight on the joint. However, patients need to change their diet not only by focussing on losing weight but also because this has the potential to mitigate pro‐inflammatory mediators, including cytokines, interleukins, histamine and free radicals that lead to increasing systematic inflammation.^52^, ^53^, ^54^, ^55^, ^56^ Patients should be made aware of the fundamental role of diet in their care process and should be guided to no longer see it as a liminal treatment.

The functional aspect can be defined as the basic expectations about how care is delivered, and it comprises, among others, the delivery of effective treatments by trusted professionals and the coordination and continuity of care.^17^, ^37^ When considering the former, the interviewees did not perceive that the indications they had received were based on evidence, and this raised a general sense of uncertainty. Uncertainty is a poorly addressed and managed issue in healthcare, which can result in patients' poor or inefficient coping strategies and dysfunctional adaptation to illness, as well as in a conflicting relationship with health professionals.^57^, ^58^, ^59^ For what concerns the latter, the interviewees did not perceive the coordination and continuity of care as smooth and clear. In fact, the interviewees felt frustrated and angry, as they did not receive straightforward treatment. This was also powered by the fact that the different health professionals they had met had different and incoherent opinions, and therefore they perceived that indications about the OA decision‐making process were based on health professionals' preferences and attitudes.

Patients desire empowerment and are keen to be actively involved in their own care process.^42^ If they do not find answers to their questions from a source (e.g., their health professional), they will seek them through other outlets, such as different professionals, acquaintances or the Internet.^42^, ^60^ This is in line with what was observed in our study, where the sense of uncertainty experienced by the interviewees led them to seek information from different professionals, the Internet and word‐of‐mouth. However, as pointed out by Chou et al.,^42^ the patients' necessity to interrogate various sources can indeed stem from the dissatisfaction with the information retrieved from one source, but it can also arouse from the patients' need to gather information from different and complementary sources to receive a tailored and holistic approach. In line with this, there rises a need to create ad hoc resources for people with OA whose content is based on solid evidence and that is tailored to their needs and expectations.

The relational aspect concerns all interactions between patients and health professionals.^37^ The interviewees felt as if they were not being understood by both their relatives and health professionals, and they found a lack of empathy in the professionals they met. Empathy declines throughout medical formation, especially when it is not trained with specific interventions.^61^, ^62^ Treatments devoid of empathy spoil the relationship between patients and health providers, and may lead to dissatisfied patients who are in turn discouraged to stick to the recommended interventions, resulting in poor health‐related outcomes.^63^ An empathetic communication style is a fundamental skill that health professionals need to learn and optimise because it can allow patients to reach good compliance towards treatments and, therefore, better outcomes. A strategy reported by patients for an efficient communication style was the use of metaphor to explain the pathology. Human beings rely on metaphor to understand the world around them. The use of relevant metaphors that tap into the patients’ life and experience can ease the creation of a bond of trust between the health professional and the patient, leading to a truly therapeutic alliance,^64^, ^65^ which is fundamental when the care process of patients need a high level of patients’ compliance, as in the case of OA.

In particular, this lack of empathy was found during the diagnosis and the prognosis of OA. The individuals interviewed extracted a sense of hopelessness from health professionals' words, which was derived from seeing OA as a pathology with a sealed faith—surgery—and a sign of their ageing process. This was also fostered by the reading of their radiographic findings that physicians found necessary to perform the diagnosis and commented with sentences such as “if I did not know that these x‐rays belonged to you, I would think that they belong to someone who is at least 30 years older than you”. However, radiographic findings are considered by CPGs, as complementary for the assessment of OA and should be considered only when other diseases are the suspected cause of the symptoms (e.g., infection, cancer, rheumatoid arthritis) or once the surgical intervention has been planned.^8^ Besides, there is a weak association between the severity of radiographic findings, pain and disability levels ^28^, ^66^ and basing clinical decisions on imaging fosters the perception of OA as a wear‐and‐tear disease, which may, in turn, induce fear‐avoidance behaviours.^28^, ^67^ Finally, promoting surgery as the unique and real solution to OA may demotivate patients to change their life habits, considering the difficulty to follow diet and a physical activity programmes compared to the ‘easy way out’ of undergoing surgery.

Some limitations of this study need to be underlined. Firstly, the small sample size required to conduct a qualitative study limits the generalisability of the results.^68^ Besides, all the interviewees lived in a similar geographical area (i.e., northern Italy), so that it is not possible to conclude that the results of this study may be transferrable to the other Italian regions. However, in Italy, there is a well‐known negative gradient gap from North to South when it comes to the efficiency of the healthcare system.^69^, ^70^ In light of this, it is possible that this study depicted the best‐case scenario that patients with OA may experience. Moreover, the results gathered from this study may not be transferrable to other European countries, such as the ones comprised in the northern areas, due to the geopolitical differences between them.^71^ Conversely, they may be more applicable to other Mediterranean countries, considering the similarities in the health professionals' educational needs within the field of rheumatology.^71^ Secondly, the patients interviewed were at different stages of their care process, and their experience may change during the different stages. However, all patients agree with what emerged during the member checking phase. On the contrary, one of the strengths of this study is that it is the first one that takes into account the whole care process experienced by patients with OA from diagnosis to pre‐surgery, highlighting some of the possible pitfalls that both patients and clinicians may encounter, and that can hinder the success of the intervention.

## 5 CONCLUSIONS

This study highlights potential common themes in the experience of people with OA, with a focus on its care process, which should be taken into account to enhance the quality of it. People with hip and knee OA seem to experience an uncertain care process. In particular, they experienced a lack of clear explanations, a lack of empathy, and a general, negative attitude towards first‐line nonsurgical treatments. All those factors underline the importance of providing patients with adequate information through effective communication about the treatment options. By doing so, it will be possible to shift patients’ beliefs and improve their awareness of the first‐line treatments they should follow. This will enhance patient‐centred treatments led by shared decision‐making processes with patients, increasing their compliance towards first‐line interventions and their skills to take care of their health and healthcare, with positive effects on their health‐related outcomes and healthcare costs.

## CONFLICTS OF INTEREST

The authors declare no conflicts of interest.

## AUTHOR CONTRIBUTIONS

Simone Battista made substantial contributions to the conception or design of the work, the acquisition, analysis or interpretation of data. Mattia Manoni made substantial contributions to the conception or design of the work, the acquisition, analysis and interpretation of data. Andrea Dell'Isola made substantial contributions to the conception or design of the work, analysis, and interpretation of data. Mattia Manoni made substantial contributions to the conception or design of the work, and interpretation of data. Alvisa Palese made substantial contributions to the design of the work, the acquisition, analysis or interpretation of data. Marco Testa made substantial contributions to the conception and design of the work, the acquisition, analysis or interpretation of data. All authors drafted the work or revised it critically for important intellectual content. All authors approved the version to be published. All authors agreed to be accountable for all aspects of the work in ensuring that questions related to the accuracy or integrity of any part of the work are appropriately investigated and resolved.

## ETHICS STATEMENT

Ethics approval was obtained from the Ethics Committee for University Research (CERA: Comitato Etico per la Ricerca di Ateneo), University of Genova (Approval date: 15 June 2020; CERA2020.07). The participants signed informed consent before participation.

## Supporting information

Supplementary information.

## Data Availability

Data are available upon reasonable request to the corresponding author.
